# Nursing performance and occupational risks in family health strategy: an integrative review

**DOI:** 10.47626/1679-4435-2022-798

**Published:** 2023-08-08

**Authors:** Francisco Werbeson Alves Pereira, Douglas Vieira Braga, Samyra Paula Lustoza Xavier, Moziane Mendonça de Araújo, Rosely Leyliane dos Santos, John Carlos de Souza Leite

**Affiliations:** 1 Universidade Regional do Cariri, Iguatu, CE, Brazil; 2 Enfermagem, Hospital Regional de Iguatu, Iguatu, CE, Brazil

**Keywords:** primary health care, nursing team, family health strategy, occupational risks, worker’s health, atenção primária à saúde, equipe de enfermagem, estratégia saúde da família, riscos ocupacionais, saúde do trabalhador

## Abstract

Primary health care is one of the different fields of nursing practice. The COVID-19 pandemic has caused a series of questions regarding exposure that nursing professionals face in terms of occupational risks in primary health care. The aim of this study was to discuss to discuss, based on scientific evidence, the occupational risks experienced by the nursing team in the Family Health Strategy. This integrative literature review was conducted in the Latin American and Caribbean Health Sciences Literature (LILACS) database and the Nursing Database (BDENF) between June and October 2020. The descriptors used in the search were: nursing, occupational risks, and primary health care. Inclusion criteria were original, open access articles published between 2005 and 2019 with the full text available. Of the 37 articles initially found, 6 were included in the analysis and after screening. Despite their presence during nursing work, occupational risks are not very perceptible except for biological risks, the understanding of which comes from professional practice rather than research and/or training. No article was addressed about the risk map or its use in the Family Health Strategy. Health managers must offer personal protective equipment and establish biosafety standards and training activities about occupational risks.

## Abstract (pt)

Entre os diversos campos de atuação da enfermagem, há a área de atenção primária à saúde. A pandemia causada pelo novo coronavírus ocasionou uma série de indagações referentes à exposição em que se encontram os profissionais de enfermagem perante os riscos ocupacionais na atenção primária à saúde, mais especificamente na estratégia saúde da família. Neste estudo, objetivou-se discutir, a partir de evidências científicas, os riscos ocupacionais vivenciados pela equipe de enfermagem na estratégia saúde da família. Tratou-se de uma revisão integrativa da literatura, realizada na base de dados Literatura Latino-Americana e do Caribe em Ciências da Saúde (LILACS) e na Base de Dados de Enfermagem no período de junho a outubro de 2020. Os descritores estabelecidos para busca foram enfermagem, riscos ocupacionais e atenção primária à saúde. Os critérios de inclusão foram pesquisas no formato de artigo original, disponíveis em formato completo e gratuito, publicadas entre 2005 e 2019. Inicialmente, foram encontrados 37 estudos; após o processo de triagem, permaneceram seis artigos. Apesar de presente durante a atuação do trabalho de enfermagem, os riscos ocupacionais ainda não são tão perceptíveis, com exceção do risco biológico, cujo entendimento é oriundo da prática profissional, e não de pesquisas e/ou capacitações. Nenhum artigo abordou o mapa de risco ou a sua utilização na estratégia saúde da família. Existe a necessidade de que os gestores em saúde tenham sensibilidade em ofertar equipamentos de proteção individual, estabeleçam normas de biossegurança e promovam atividades que visem à capacitação desses profissionais sobre riscos ocupacionais.

## INTRODUCTION

Since the Brazilian Unified Health System was created in 1988, the health situation of Brazilians has changed, and the possibilities for professional nursing activities have expanded significantly. Since that time, the profession’s core axis has become the planning, implementation, and evaluation of policies and actions in the field of public and collective health, bringing greater resolution, quality, and efficiency to the provided care, from large centers to the most remote locations in the country.^1,2^

Among the contexts of action, the Family Health Strategy (Estratégia Saúde da Família: ESF), a gateway to primary health care, characterizes the essence of nursing work as the promotion, protection, maintenance, and rehabilitation of the health of individuals and families and the community based on care, management, and educational actions, which must conform to the principles and assumptions of the Unified Health System, as well as to the demands of the population.^2^

The work environment has a significant influence on quality of life and health,^3^ As the complexity of nursing work in the ESF has increased, it is clear that these professionals have increasingly become the anchor of primary health care, and concern about the occupational risks to which they are exposed has also increased. Several ESF activities expose nursing professionals to risks, including vaccination, wound dressing, cervical smear, heel prick test, reprocessing of personal items, waste disposal, provision of care in the home environment, etc.^1,2^ Interpersonal conflicts, work overload, occupational stress, and exposure to social vulnerabilities, such as violence, can impact their physical and mental health.

Prolonged exposure to occupational hazards contributes to accidents at work.^4^ In 2018 in Brazil, there were 2,472 reports of occupational exposure to risk factors and 576,951 notifications of work accidents, of which 71,496 were related to human health care activities.^5^ Despite such data, scientific production on occupational risks is still incipient, and the number of publications has not increased significantly over time.^6^

However, the pandemic has added COVID-19 to the list of work-related diseases^7^ and highlighted the work of the nursing team, focusing debate about the occupational risks to which they are exposed. However, these discussions are mainly about hospital-level care practices^8,9^; debate about the risks to which ESF professionals are exposed must be expanded.^10^

The present study’s relevance is to stimulate discussion about the occupational risks to which nursing workers in the ESF are exposed, since it can indicate measures to help guarantee injury prevention and promote the safety and health of these workers. Based on the above, this study aims to discuss, based on scientific evidence, the occupational risks experienced by nursing teams in the ESF.

## METHODS

Mendes et al.^11^ considered the following steps for developing a literature review: define the guiding question, search for and select primary studies, extract the data, assess the included studies, interpret the results, and present the review. This review was conducted between June and October 2020. The research question was developed using the Population, Variable, and Outcomes framework,^12^ which determined its components and the corresponding descriptors, as shown in Table 1.

**Table 1:** Descriptors used in the research question according to the Population, Variable and Outcomes strategy

	Elements	Descriptor
P - Population	Nurses	Nursing
V - Variable	Occupational risks	Occupational risks
O - Outcomes	PHC work	PHC

PHC = Primary health care.

Thus, the following research question was defined: “What occupational risks are nursing professionals exposed to while working in primary health care?” The search was performed in the Latin American and Caribbean Health Sciences Literature (LILACS) database and the Ministry of Health’s Nursing Database (BDENF). These databases were selected due to their relevance in the academic and scientific environment and because they contained the highest number of documents related to the study. The following descriptors (in Portuguese) were used: *enfermagem*/nursing, *riscos ocupacionais*/occupational risks, and *atenção primária à saúde*/primary health care.

The inclusion criteria were original, open access articles published between 2005 and 2019 with the full text available. This time frame was selected since it coincided with the passing of worker safety Regulatory Norm 32 on November 11, 2005. This legislation, which established basic guidelines for measures to protect the health and safety of health service workers,^13^ was an important milestone in occupational health in Brazil, stimulating discussion about the subject. The exclusion criteria were review articles, monographs, dissertations, theses, and studies that did not consider the occupational health of nursing professionals in the primary health care context.

**Figure 1:**
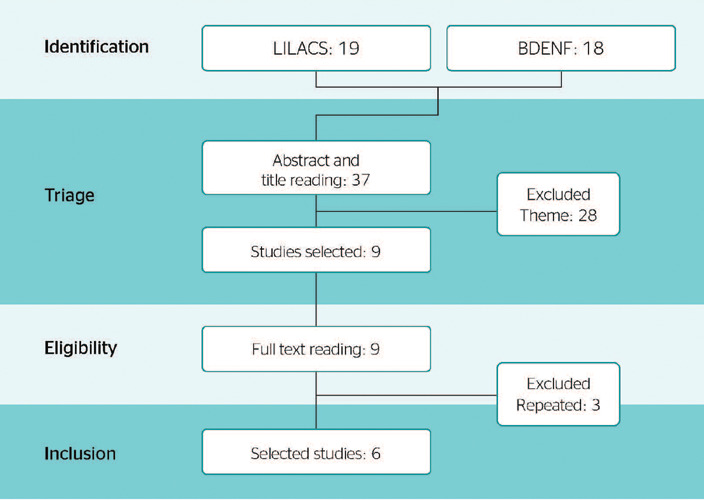
Article search and selection process. BDENF = Base de Dados de Enfermagem (Nursing Database); LILACS = Latin American and Caribbean Health Sciences Literature.

The database searches were conducted by cross-referencing the descriptors with the Boolean operator “AND”. Initially, 37 studies were identified: 19 in LILACS and 18 in BDENF. The first stage of the screening process involved title and abstract reading, in which 28 studies were excluded due to lack of relevance (15 from LILACS and 13 from BDENF). In the second phase of screening, the full texts of the 9 remaining studies were read, 3 of which were duplicate publications and were excluded. Thus, 6 studies met the eligibility criteria and were included in the analysis. The study selection process, which followed the Preferred Reporting Items for Systematic Reviews and Meta-Analysis checklist, is presented in Figure 1.

Data extraction was based on an instrument by Ursi,^14^ which was adapted to collect the variables of interest: (1) bibliographic aspects, such as authorship, title, study type, year of publication, and country, and (2) occupational risk aspects involved in nursing work in the primary health care context. The study evaluation stage was based on the pyramid of scientific evidence proposed by Galvão,^15^ which indicated that all 6 included studies are descriptive, thus ranking at the sixth level.

The results were organized based on a summary of the findings, which allowed the analysis and interpretation of the data by identifying converging and diverging aspects, which are presented below descriptively.

## RESULTS AND DISCUSSION

It should be pointed out that only two articles, David et al.^16^ and Santos et al.,^17^ described the professional profiles of the authors, and only Santos et al.^17^ included an occupational health specialist. The samples of only 2 articles consisted exclusively of nurses.^17,18^ The others aimed to encompass all nursing professionals, ie, nurses and nursing technicians/assistants.^16,19,20,21^ Table 2 presents the titles, authors, objectives, study types, samples, country, and year of publication of the 6 articles included in this review.

**Table 2:** Synthesis of the included studies

Authors	Title	Objective	Study type	Sample	Country/year
Arcanjo RVG, Chistovam BP, Braga ALS, Silvino ZR^18^	Gerenciamento dos riscos ocupacionais da enfermagem na atenção básica: estudo exploratório descritivo	To identify the occupational risks to which nursing professionals are exposed in primary health care; to describe the risk factors present in health units and correlate them with safety conditions.	Exploratory and descriptive study with a quantitative approach	8 nursing professionals	Brazil/2018
Melo FMS, Oliveira BB, Oliveira RKL, Bezerra JC, Silva MJN, Joventino ES^20^	Conhecimentos de enfermeiros sobre acidentes de trabalho	To determine nurses’ knowledge about work accidents.	Qualitative research	10 nurses	Brazil/2017
De Sousa ÁFL, Queiroz AAFLN, Oliveira LB, Moura ME, Batista OMA, Andrade D^19^	Representações sociais da enfermagem sobre biossegurança: saúde ocupacional e o cuidar prevencionista	To understand the social representations of biosafety among nursing professionals in primary care and analyze how they are associated with care quality.	Exploratory study with a qualitative approach	36 nursing professionals	Brazil/2016
Silva CCS, Rodrigues LMC, Silva VKBA, Silva ACO, Silva VLA, Martins MO^21^	Percepção da enfermagem sobre condições de trabalho em unidades de saúde da família na Paraíba – Brasil	To describe and characterize the perceptions of nursing professionals about accidents and work conditions at family health units in João Pessoa, Paraíba.	Descriptive study with a qualitative approach	12 nursing professionals	Brazil/2013
Santos SR, Virgolino JLB, Brito SS, Bezerra EP, Dantas UIB, Costa MML^17^	Risco ocupacional enfrentado pelos enfermeiros que atuam na atenção primária à saúde	To analyze the perceptions of primary health care nurses about biosafety measures in their work process.	Descriptive and exploratory study with a quantitative and qualitative approach	53 nurses	Brazil/2013
David HMSL, Mauro MYC, Silva VG, Pinheiro MAS, Silva FH^16^	Organização do trabalho de enfermagem na atenção básica: uma questão para a saúde do trabalhador	To determine the impact of the work organization of nurses, technicians, and nursing assistants in primary care and its relationship with their health.	Quantitative, cross-sectional, descriptive, and inferential study	171 nursing professionals	Brazil/2009

Occupational hazards are classified into 5 categories: physical, chemical, biological, ergonomic, and mechanical/accidental.^16,17,18,19,20,21^ Among occupational risks in the context of primary health care, the biological type was the most discussed in the included studies, mainly due to the handling of biological agents, eg, contaminated needles and sharp objects. However, the high biological risk among nurses is not only due to contaminated sharp objects, but to contact with blood, tissue, and body fluids while performing their activities.^18,19^

This review showed that biological risk is responsible for most of the work accidents in the ESF.^17^ Thus, it is common for professionals to only associate work accidents with biological risk because it is the most common and visible type in their professional activities, as well as due to the lack of training they receive about it.

Physical risk is present mainly in home visits, during which professionals either do not use or are not provided sunscreen, which requires managers to be more committed to their protection.^19^ Mechanical risk is related to falls, physical aggression, stress, pressure from patients, and job dissatisfaction. Chemical risk is mainly related to medications. Ergonomic risks are related to walking, repetitive movements, and inadequate workplace furnishings.^18^

Another point worth mentioning is that violence and psychosocial risks are not directly covered in the Regulatory Norms, although they appear separately within other topics or are included in mechanical risks.^18^ None of the studies addressed the risk map or its importance in health services, which would be essential for more comprehensive identification of occupational risks and their causes for nursing professionals in the ESF.

Studies indicate that this theme has a higher prevalence at the hospital level, which exposes a weakness by researchers and professionals in the ESF context, ie, the constant division between actions of a curative nature and those of health promotion and risk control. Thus, the greatest difficulty is for researchers and health professionals to understand that work accidents and occupational risks are inherent to any level of health care, including the ESF.^16,20^

Knowledge of the occupational risks to which workers are exposed in the ESF is one of the main ways of preventing accidents. Health managers must devise strategies to improve working conditions and change the behavior of the nursing team, which can be achieved through permanent education programs.^21^

Preparation about the types and definitions of occupational risks is still inadequate.^18^ The knowledge that these professionals have about occupational risks comes from daily practice, not from research and/or qualification/training. This can lead to misidentification of risk or ineffective prevention measures.^18^

Another factor that entails risk is unhealthy working conditions related to the unavailability of equipment and instruments, financial hardship, and instability of the employment relationship, which predisposes workers to physical, ergonomic, and psychosocial occupational risks, thus increasing their vulnerability in the workplace. It is important to highlight that, even when personal protective equipment is provided, training must also be provided and awareness raised.^17,21^

Some factors influence the inappropriate use of personal protective equipment, such as discomfort or inconvenience, carelessness, forgetfulness, lack of habit or discipline, inadequate equipment, insufficient quantity, and ignoring it because it seems unnecessary. New meaning must be given to the continuous education process, connecting it with occupational health, thus enabling the proper use of personal protective equipment.^21^

Therefore, it is necessary to follow the recommendations of the NRs. The main NR dealing with health professionals is Regulatory Norm 32, which delineates measures to protect their safety and health. In this context, the use of personal protective equipment, hand hygiene, vaccination against hepatitis B, tetanus, and diphtheria, etc., are recommended. According to Regulatory Norm 32, it is the duty of health services to provide personal protective equipment in adequate quantity and quality, in addition to being responsible for its conservation. Another important point is that workers must report any changes that makes the equipment unsuitable for use.^17,21^

Actions such as occupational risk training and the establishment of prevention and notification measures can lead to a considerable reduction in work accident rates in the ESF. However, correct reporting of work accidents due to risk exposure is still a major challenge, since a lack of knowledge contributes to underreporting.^20^

Since notification is the main means of learning about work-related accidents and illnesses, it is critical for the development of risk prevention and control strategies. However, the perceived importance of notifying occupational health is still weak among nursing professionals. Incomplete implementation of occupational health policies in the ESF impacts these workers, who end up being unaware of risk issues and work accidents.^21^

It is known that strategies to prevent or reduce the occupational risks to which nursing professionals are exposed guarantees an adequate work model. Such strategies are mainly implemented through policies, standard operating procedures, and the control and assessment of risks and adverse events that affect safety, health, professional integrity, the environment, and the institution’s image.^18^

## CONCLUSIONS

The results of this study show how incipient scientific production is on the occupational risks to which nursing professionals are exposed while performing their ESF duties. It also allowed an overview of health services management in this context, since these professionals often do not have the support or training they need to identify or deal with work accidents due to occupational risks.

It is important to point out that the mitigating factors for occupational risks are mainly associated with management, which is responsible for training these professionals on the subject. Therefore, health managers should be more sensitive to the issue, offering training courses, permanent education, and subsidies for these professionals to carry out their activities safely, thus ensuring that public policies are effective.

Although this study contributes to the field of occupational health among nursing professionals, it involves certain limitations, such as scarce literature about the theme and studies that focus on specific regions of the country, that, while hindering generalization of the findings, also serve as recommendations for more in-depth studies about this situation.

## References

[1] Da Silva MCN, Machado MH (2020). Sistema de saúde e trabalho: desafios para a Enfermagem no
Brasil. Cienc Saude Colet.

[2] Ferreira SRS, Périco LAD, Dias VRFG (2018). A complexidade do trabalho do enfermeiro na Atenção
Básica à Saúde. Rev Bras Enferm.

[3] Sampaio CL, De Almeida PC, Alves e Souza AM, Neri MFS, Da Silva LA, Caetano JÁ (2020). Diferenças entre qualidade de vida e enfrentamento ocupacional de
enfermeiros efetivos e terceirizados. Rev Bras Enferm.

[4] Fonseca EC, Sousa KHJF, Nascimento FPB, Tracera GMP, Dos Santos KM, Zeitoune RCG (2020). Riscos ocupacionais na sala de vacinação e suas
implicações à saúde do trabalhador de
enfermagem. Rev Enferm UERJ.

[5] Ministério da Fazenda (2018). Anuário Estatístico de Acidentes do Trabalho: AEAT 2017. Vol. 1
(2009).

[6] Chiodi MB, Marziale MHP (2006). Riscos ocupacionais para trabalhadores de Unidades Básicas de
Saúde: revisão bibliográfica. Acta Paul Enferm.

[7] Silva LS, Machado ElL, De Oliveira HN, Ribeiro AP (2020). Condições de trabalho e falta de informações
sobre o impacto da COVID-19 entre trabalhadores da saúde. Rev Bras Saude Ocup.

[8] Belarmino AC, De Mendonça KM, Rodrigues MENG, Ferreira Junior AR (2020). Saúde ocupacional da equipe de enfermagem obstétrica
intensiva durante a pandemia da Covid-19. Av Enferm.

[9] Dias CVP, Damasceno JC, Silva LVF, Da Rocha BM (2020). Saúde do profissional de Enfermagem: riscos ocupacionais em ambiente
hospitalar. Saúde (Santa Maria).

[10] Sudo RA, Carvalho IVC, Bonett OP, De Oliveira A, Miranda MAL, Göttems LBD Proteção e biossegurança dos profissionais de enfermagem da
atenção básica no contexto da COVID-19.

[11] Mendes KDS, Silveira RCCP, Galvão CM (2008). Revisão integrativa: método de pesquisa para a
incorporação de evidências na saúde e na
enfermagem. Texto Contexto Enferm.

[12] De Campos LD, Ferraz RRN (2015). A prática do enfermeiro clínico na assistência aos
usuários da Estratégia Saúde da Família: síntese de
evidências. Rev UNILUS Ensino Pesqui.

[13] Brasil, Ministério do Trabalho e Emprego (2005). Portaria nº 485, de 11 de novembro de 2005. Aprova a norma regulamentadora nº 32
(Segurança e saúde no trabalho em estabelecimentos de
saúde).

[14] Ursi ES (2005). Prevenção de lesões de pele no perioperatório:
revisão integrativa da literatura.

[15] Galvão CM (2006). Níveis de evidências [editorial]. Acta Paul Enferm.

[16] David HMSL, Mauro MYC, Silva VG, Pinheiro MAS, Da Silva FH (2009). Organização do trabalho de enfermagem na
Atenção Básica: uma questão para a saúde do
trabalhador. Texto Contexto Enferm.

[17] Santos SR, Virgolino JLB, Brito SS, Bezerra EP, Dantas UIB, Costa MML (2013). Risco ocupacional enfrentado pelos enfermeiros que atuam na
atenção primária à saúde. Rev Enferm UFPE Online.

[18] Arcanjo RVG, Chistovam BP, Braga ALS, Silvino ZR (2018). Gerenciamento dos riscos ocupacionais da enfermagem na
atenção básica: estudo exploratório
descritivo. J Res Fundam Care.

[19] De Sousa ÁFL, Queiroz AAFLN, De Oliveira LB, Moura MEB, Batista OMA, De Andrade D (2016). Representações sociais da Enfermagem sobre
biossegurança: saúde ocupacional e o cuidar prevencionista. Rev Bras Enferm.

[20] Melo FMS, De Oliveira BSB, De Oliveira RKL, Bezerra JC, Silva MJN, Joventino ES (2017). Conhecimentos de enfermeiros sobre acidentes de trabalho. Rev Rene.

[21] Silva CCS, Rodrigues LMC, Da Silva VKBA, De Oliveira e Silva AC, Do Amaral e Silva VL, Martins MO (2013). Percepção da enfermagem sobre condições de
trabalho em unidades de saúde da família na
Paraíba-Brasil. Rev Eletr Enf.

